# Can Gamification Contribute to Computer Modeling-Driven Biomedical Research?

**DOI:** 10.3389/fphys.2018.00908

**Published:** 2018-07-19

**Authors:** Julio Vera, Guido Santos

**Affiliations:** Laboratory of Systems Tumor Immunology, Department of Dermatology, Universitätsklinikum Erlangen and Faculty of Medicine, Friedrich-Alexander University Erlangen-Nürnberg, Erlangen, Germany

**Keywords:** gamification, mathematical and computational modeling, biomedicine, pneumonia, games, crowdsourced science, citizen science

Not all games have entertainment as their principal objective. “Serious games” combine entertainment with more productive purposes, including furthering the public understanding of scientific concepts and promoting scientific research conducted by amateur scientists (Aldrich, 2009; Deterding et al., 2011b). Serious games can explain, in a simplified manner, molecular or cellular processes involved in the onset of diseases such as rheumatoid arthritis (https://goo.gl/LRE2AS). Other serious games are employed to support conventional methods of diagnosis; they include games in which the patient's performance is measured in order to be correlated with, for instance, the onset of neurodegenerative disease (Morgan, 2016). Finally, the gaming experience can serve the analysis of real scientific data by exploiting the ability of the gamer's brain to perform complex pattern recognition tasks (Luengo-Oroz et al., 2012). One notable example of this kind is *Foldit* (https://fold.it), a game which requires trained gamers to identify the 3D structure of proteins by rotating and interactively manipulating their unfolded structures. Khatib *et al*. have shown that heuristics based on the strategies followed by outstanding *Foldit* players outperformed standard computational methods for protein structure discovery (Khatib et al., 2011). More recent examples of similarly game-based research strategies are *Play to Cure: Genes in Space* (Coburn, 2014), a game designed to crowdsource genomic data analysis, and *Eyewire*, a game whose purpose is the analysis of images of the brain (https://eyewire.org/). The application of game-based strategies to the end of solving problems in research, industry or education is called “gamification.” In general, gamification consists in the use of elements of game design in non-game contexts (Deterding et al., 2011a). One example of such contexts is crowdsourcing, an unconventional research strategy in which open calls for collaboration bring together a large community of participants for the effort to solve complex scientific problems (Schrope, 2013; Lee et al., 2017). There have been several recent successful cases of crowdsourcing applied to biomedical problems (Kawrykow et al., 2012; Luengo-Oroz et al., 2012; Loguercio et al., 2013; Good et al., 2014; Rallapalli et al., 2015). Evidence suggests that gamification makes crowdsourcing more appealing to contributors (Bowser et al., 2013; Eveleigh et al., 2013; Hamari et al., 2014). Accordingly, this paper will discuss the use of gamification in the solution of advanced scientific problems. Our view is that scientific tasks calling for pattern recognition in biomedical data are ideal for game-based crowdsourcing because the brain's innate abilities in pattern recognition are currently—as yet—able to compete with advanced computer algorithms (von Ahn et al., 2008). We further believe that this strategy may be productive in combination with biomedical computer simulations.

Over the last decade, mathematical and computational model simulations emerged under the “Systems Biology” paradigm as a valuable tool for the integration of biomedical data linking tissue, cellular and intracellular processes in relation to the pathogenesis of complex diseases. Such simulations can serve to detect biomarkers for disease prognosis (Khan et al., 2017), analyze the feasibility of treatments or predict the individualized course of a disease (Passante et al., 2013), or find new drug targets (Vera et al., 2007). This notwithstanding, there are some circumstances in which computational methods struggle to identify the regulatory patterns underlying the onset of some diseases as predicted by simulations.

In response to this challenge, we have analyzed a mathematical model accounting for the early phases of pneumonia infection that we developed within a project on medical systems biology (https://www.capsys.imise.uni-leipzig.de/en); the original article inspiring the discussion in this paper is that of Santos et al. (2018). Pneumonia is a highly prevalent inflammatory infection of the lungs that causes millions of deaths each year, especially in individuals with immature or compromised immune systems such as children and elderly people (McIntosh, 2002). The most common cause is bacterial infection (Kochanek et al., 2016). In contrast to other inflammatory diseases, the resolution of the infection is time-critical, because the inflammation inundates the lung alveoli with liquid and impedes proper ventilation, often with fatal effects. The optimum treatment would be prevention, and although vaccination against pneumococcal bacteria is an available option, its efficacy is low in children and elderly people (Sjöström et al., 2006; Nguyen et al., 2017), leading to greater exposure to the disease among populations whose immune systems it most severely challenges.

In order to better understand at the microscopic level the very first stages of alveolar infection and suggest therapeutic strategies for the prevention of lung infection and inflammation, we used computational modeling and simulation (Cantone et al., 2017; Schulz et al., 2017; Santos et al., 2018). The mathematical model we developed is multi-level in the sense that it integrates processes describing cell-to-cell interactions between the bacteria, the immune cells and the lung epithelial cells with a description of the intracellular processes governing these cells. When simulating the model, we realized that detecting the differences between relevant biomedical scenarios, for instance resolved versus progressing infection, required the performance and classification of thousands of 2D and 3D computer simulations and the identification of spatial patterns (see Figure 1A). A “pattern,” in the context of our model, is defined as a spatial distribution of the elements of the model (bacteria, macrophages, chemokines) through the environment (the inner alveolus surface). We need to identify and classify these patterns because groups of patterns from the model can be correlated with different infection scenarios, such as pneumonia acquired in the hospital or in the context of an epidemic. The conventional approach to tackling this task would be to process the simulations, eliminate invalid or irrelevant ones, and classify those remaining, followed by the application of multivariate statistical analysis techniques (Santos et al., 2016; Nikolov et al., 2018). Our hypothesis here is that it is possible to crowdsource the analysis of the patterns in our model simulations of lung infection. There is at least one example in the literature in which crowdsourcing has been proposed for the analysis of biomedical mathematical simulations (http://cancercrusadegame.com/). Here, we argue that transforming the computational model into a serious game can increase the chances of success in crowdsourcing.

**Figure 1:**
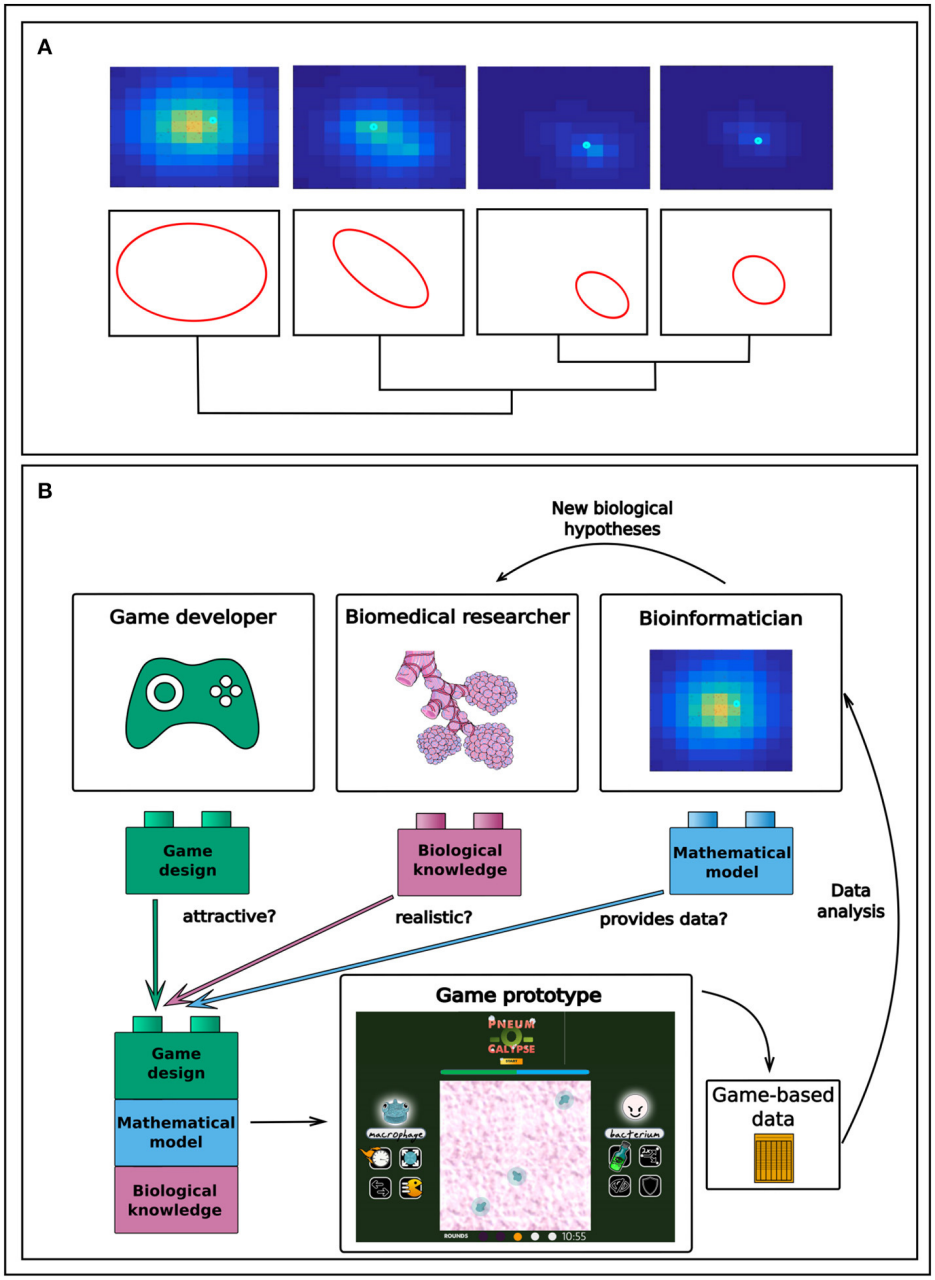
**(A)** Pattern recognition applied to spatial simulations can provide a tool for the classification of various infection phenotypes. Here, differences in bacteria infectivity (red dots, bacteria) and inflammation (yellow gradient, chemokines as surrogates of inflammation) are visualized as different patterns of color in a computer simulation of an infected lung alveolus. (**B)** Workflow of a gamification-inspired research project in biomedicine, created on the basis of the results from our experience in the hackathon about serious games in lung infection (http://gaminfectionhack.weebly.com). Biomedical and translational researchers contribute biomedical knowledge, on which bioinformaticians build to add the computational model. On this basis, game designers then create a game attractive to the intended audience. The gaming experience of the players is stored and subsequently mined by the bioinformaticians to construct hypotheses such as that on the bacteria phenotypes influencing infection resolution.

We ran a hackathon in Erlangen, Germany, on the last weekend of September 2017, and published the results online on the event's website (http://gaminfectionhack.weebly.com). The hackathon's purpose was to explore the feasibility of transforming into a serious game our computational model describing the initial phase of bacterial lung infection, and to sketch and prototype serious games on the basis of our computational model. About 40 people from several European countries participated in the event, including computational biologists, clinicians, biomedical researchers, microbiologists, designers, programmers and even high school students with game programming skills. We organized the participants into multidisciplinary teams. The hackathon produced a selection of game prototypes about lung infection. All of them are playable and appear on the event's website (http://gaminfectionhack.weebly.com/games.html). For instance, one of the teams proposed a two-player strategy game making use of the model simulations (Figure 1B), in which the players can play as bacteria invading a lung alveolus or as a macrophage defending the alveolus. In every turn, each player decides on the use of any of the phenotypes that macrophages or bacteria can activate during the infection, incorporated into the game as actionable “powers” of the game characters. Between turns, the game evolves using the gamer's decisions, which act as input variables in the computer model simulations. An improved version of this game might store and analyze the gaming experience of a large number of players in order to identify, for example, preferential sequences of bacteria phenotypes that have to be triggered during the simulation to generate a productive infection.

In engineering, we try to perform a task following the most efficient strategy. By contrast, according to Suits, games are based on preventing the most efficient approach being *the unique option* by defining a set of operative restrictions such that the users can come up with and follow alternative strategies that they would not follow outside the gaming setup (Suits, 1967). In other gamification approaches, such as those applied in *Foldit* or *Eyewire*, the game developers, rather than employing this strategy, have designed user-friendly platforms for crowdsourced data analysis in which sets of tasks are proposed to the users. Our suggestion here is that it is possible to create actual games which encode biologically inspired mathematical models for the definition and execution of the game's rules. In this sense, the mathematical model defines the sets of restrictions within which the game takes place and the user's creativity has to evolve.

We argue that the technology required to perform this approach is available, and that multiple examples in other related fields, such as bioinformatics, imaging and structural analysis (cf. the previously mentioned *Foldit* and *Eyewire*), suggest that crowdsourcing can also contribute to biomedical simulation data. Designing games suitable for this purpose would entail the creation of multidisciplinary teams composed of game developers, translational researchers and bioinformaticians (Figure 1B). In an interactive ideation process, the translational researchers would contribute their profound knowledge around the biomedical problem, the bioinformaticians would analyze the usable data generated out of the game simulations, and game developers would work on transforming the game concept into an enjoyable and attractive experience for the audience. Generating scientific insights would call for storage of the data output generated during the gaming experience, which the bioinformaticians and translational researchers would subsequently analyse. In our opinion, the bottleneck occurs in the ability to convert a computational model into a game that provides both a satisfactory gaming experience and data that are useful from a biomedical perspective. Our experience in the hackathon indicates that achieving this combination is possible where multidisciplinary teams are put in place.

We do, however, perceive the continued presence of obstacles to the development of this approach. The principal limitation consists in a lack of sufficient numbers of encouraging case studies in which this strategy has rendered valuable biomedical insights. We believe that this is due, alongside the intrinsic novelty of the approach, to the scarcity of funding resources that could eventually support projects following this approach.

Computational model-inspired games can generate benefits for the user as well as for the researcher, and can thus serve a secondary but important educational purpose; they may, for instance, train specific cognitive abilities, increase gamers' knowledge of the biomedical topic of the game, and attract users to science, or inspire them to become more involved in it (Abeele et al., 2015). In an interesting example, a game has been developed to help students train advanced surgical skills. This game, together with other related examples of serious games for medical education are reviewed in Graafland et al. (2012). The dynamic nature of games and simulations can provide students with the temporal dimension of biological processes that traditional lectures cannot deliver. Further, in contrast to video material, a game is interactive and lets the student “learn by doing.” This type of game may be a way of promoting mathematics and computer sciences in youngsters. Our hackathon illustrated the fact that gaming is no longer a purely male domain. Gamification might capitalize on the increasing interest in computer games among girls and young women to raise awareness of computational modeling in this group. Finally, simulation-based games can be excellent material for the training of students and researchers in biosciences and medicine in the abilities of computer models to formulate hypotheses or predict and assess therapies.

## Author contributions

All authors listed have made a substantial, direct and intellectual contribution to the work, and approved it for publication.

### Conflict of interest statement

The authors declare that the research was conducted in the absence of any commercial or financial relationships that could be construed as a potential conflict of interest.
